# Dementia Detection via Retinal Hyperspectral Imaging and Deep Learning: Clinical Dataset Analysis and Comparative Evaluation of Multiple Architectures

**DOI:** 10.3390/bioengineering12121362

**Published:** 2025-12-14

**Authors:** Wen-Shou Lin, Chia-Ling Chen, Shih-Wun Liang, Hsiang-Chen Wang

**Affiliations:** 1 Neurology Division, Department of Internal Medicine, Kaohsiung Armed Forces General Hospital, No. 2, Zhongzheng 1st Rd., Lingya District, Kaohsiung 80284, Taiwan; linvincent1009@gmail.com; 2 Department of Medicine, National Defense Medical University, No. 161, Sec. 6, Minquan E. Rd., Neihu District, Taipei 11490, Taiwan; 3 Department of Mechanical Engineering, National Chung Cheng University, 168, University Rd., Min Hsiung, Chiayi 62102, Taiwan; jolincchen@gmail.com (C.-L.C.); start90301@gmail.com (S.-W.L.); 4 Department of Medical Research, Dalin Tzu Chi Hospital, Buddhist Tzu Chi Medical Foundation, No. 2, Minsheng Road, Dalin, Chiayi 62247, Taiwan; 5 Director of Technology Development, Hitspectra Intelligent Technology Co., Ltd., 4F, No. 2, Fuxing 4th Rd., Qianzhen District, Kaohsiung 80661, Taiwan

**Keywords:** dementia detection, hyperspectral imaging, artificial intelligence

## Abstract

This study aimed to detect dementia using intelligent hyperspectral imaging (HSI), which enables the extraction of detailed spectral information from retinal tissues. A total of 3256 ophthalmoscopic images collected from 137 participants were analyzed. The spectral signatures of selected retinal regions were reconstructed using hyperspectral conversion techniques to examine wavelength-dependent variations associated with dementia. To assess the diagnostic capability of deep learning models, four convolutional neural network (CNN) architectures—ResNet50, Inception_v3, GoogLeNet, and EfficientNet—were implemented and benchmarked on two datasets: original ophthalmoscopic images (ORIs) and hyperspectral images (HSIs). The HSI-based models consistently demonstrated superior accuracy, achieving 84% with ResNet50, 83% with GoogLeNet, and 82% with EfficientNet, compared with 80–81% obtained from ORIs. Inception_v3 maintained an accuracy of 80% across both datasets. These results confirm that integrating spectral information enhances model sensitivity to dementia-related retinal changes, highlighting the potential of HSI for early and noninvasive detection.

## 1. Introduction

Dementia is a rapidly growing global health crisis characterized by progressive neurocognitive decline. According to recent reports, up to 45% of cases could potentially be delayed by addressing risk factors [1], yet the incidence continues to rise, with over 55 million individuals affected worldwide—a number projected to nearly triple by 2050 [2,3,4]. In Taiwan alone, the prevalence of mild cognitive impairment (MCI) and dementia in the elderly population has reached alarming levels, necessitating urgent strategies for early detection [5]. As an extension of the central nervous system (CNS), the retina shares embryological origins and physiological features with the brain, making it a promising site for non-invasive biomarker discovery [6,7]. Retinal structural and functional alterations have been shown to mirror neurodegenerative processes [8,9,10], and various ophthalmic disorders are increasingly linked to CNS pathology [11,12,13,14,15,16].

While conventional ophthalmoscopy captures structural information, it lacks the sensitivity to detect subtle biochemical changes associated with early-stage neurodegeneration. Hyperspectral imaging (HSI) addresses this limitation by capturing a “data cube” of spatial and spectral information, enabling the quantitative characterization of tissue composition [17]. A key target for HSI is amyloid-beta (Aβ), a hallmark of Alzheimer’s disease (AD). The optical detection of Aβ is grounded in light scattering theories: smaller soluble Aβ oligomers primarily induce Rayleigh scattering, whereas larger insoluble plaques, comparable in size to the wavelength of light, trigger Mie scattering [18,19,20]. These scattering phenomena alter the refractive index and spectral reflectance profile of retinal tissue, enabling HSI to identify pathological changes before gross structural damage occurs [21,22,23,24,25]. Furthermore, HSI has shown potential in assessing retinal oxygen saturation and metabolic changes, offering a multi-dimensional approach to identifying dementia biomarkers that are invisible to standard RGB imaging [26,27,28,29,30,31].

The integration of artificial intelligence (AI) with ophthalmic imaging has revolutionized diagnostic precision. Deep learning (DL) models, particularly Convolutional Neural Networks (CNNs), have achieved high accuracy in detecting diabetic retinopathy, glaucoma, and age-related macular degeneration using fundus photographs and optical coherence tomography (OCT) [32,33,34,35,36]. However, most existing AI models rely on standard color images, potentially missing the spectral nuances indicative of early pathology. By feeding high-dimensional spectral data into advanced architectures such as ResNet50 [37], Inception_v3 [38], GoogLeNet [39], and EfficientNet [40], there is an opportunity to significantly enhance diagnostic sensitivity.

This study aims to bridge the gap between spectral optics and intelligent diagnostics. We developed a deep learning framework that leverages reconstructed retinal hyperspectral images to detect dementia. To validate the clinical relevance of our model, cognitive status was standardized using the Mini-Mental State Examination (MMSE), a widely validated tool for screening cognitive impairment [41,42,43]. By comparing the performance of multiple CNN architectures on both original ophthalmoscopic images (ORIs) and hyperspectral images (HSIs), we demonstrate that integrating spectral information significantly improves the detection of dementia, particularly in distinguishing early-stage impairment from normal aging.

## 2. Materials and Methods

### 2.1. Data Collection and Study Population

**Figure 1:**
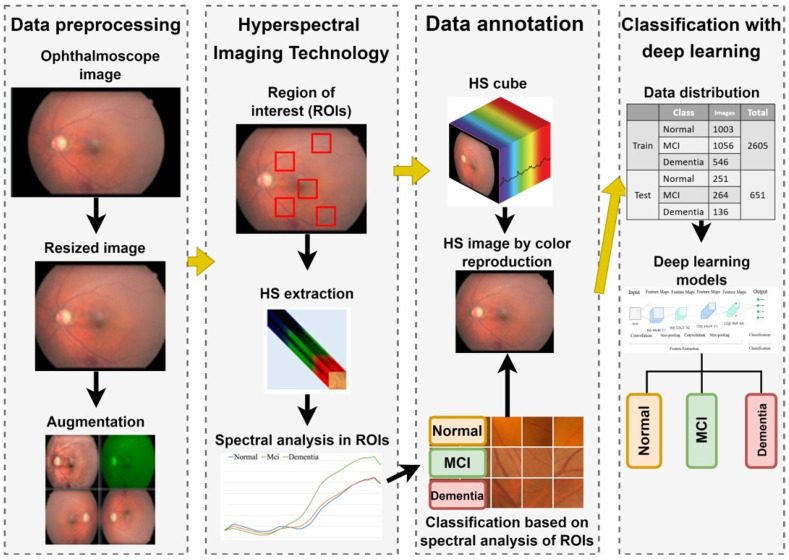
Overall experimental workflow for dementia detection using hyperspectral imaging. The process involves: (1) Acquiring fundus images and extracting spectral features from five Regions of Interest (ROIs); (2) Reconstructing hyperspectral (HS) cubes (512 × 512 × 401) from RGB images; (3) Preprocessing HS images into three-channel inputs (512 × 512 × 3) suitable for Deep Learning; and (4) Training and evaluating four CNN architectures (ResNet50, Inception_v3, GoogLeNet, EfficientNet) to classify cognitive status (Normal, MCI, Dementia) based on Mini-Mental State Examination (MMSE) scores.

The overall experimental workflow of this study is illustrated in Figure 1. This retrospective study was conducted at Kaohsiung Armed Forces General Hospital and approved by the Institutional Review Board (IRB No. KAFGH 114-025). Participants aged 60 years or older were recruited from the Neurology and Ophthalmology clinics.

Cognitive Classification:

Cognitive status was assessed using the Mini-Mental State Examination (MMSE), a validated screening tool for cognitive impairment [44,45,46,47]. Participants were categorized into three groups based on their total MMSE scores:

Normal Control (NC): MMSE 28–30

Mild Cognitive Impairment (MCI): MMSE 23–27

Dementia: MMSE 0–22

Ophthalmic Screening and Exclusion Criteria:

To ensure that spectral variations were attributable to cognitive status rather than primary ocular pathology, rigorous screening was applied. All participants underwent a comprehensive ophthalmic examination by a board-certified ophthalmologist. Patients were excluded if they presented with: Significant media opacities preventing clear fundus visualization (e.g., dense cataracts, vitreous hemorrhage). Co-existing major retinal pathologies, including high myopia (<−6.0 D), diabetic retinopathy (moderate to severe), age-related macular degeneration (AMD), or glaucoma. History of prior retinal surgery or laser therapy. Image Acquisition: Fundus images were acquired using a Kowa Nonmyd 7 retinal camera (Kowa Company Ltd., Nagoya, Japan) with a 45-degree field of view centered on the fovea. To ensure optimal image quality and standardize light entry, pharmacological mydriasis was performed on all participants prior to imaging. Images with significant motion artifacts or defocus—common in elderly patients due to poor fixation—were manually reviewed and excluded. Dataset Composition:

The final analytical sample consisted of 137 participants balanced across diagnostic categories: Normal (n = 49, 1254 images), MCI (n = 54, 1320 images), and Dementia (n = 34, 682 images). The cohort was predominantly aged 60–80 years, with a slight female predominance. Detailed demographics are provided in Table 1. A total of 137 participants were recruited for this study. From these participants, we collected data from 274 eyes. After quality control to exclude images with severe artifacts, the final dataset comprised 3256 images. The distribution was as follows: the Dementia group (including mild cognitive impairment) consisted of 77 participants (154 eyes, 1856 images), and the Normal group consisted of 49 participants (98 eyes, 1400 images).

### 2.2. Hyperspectral Reconstruction and Region of Interest (ROI) Selection

**Figure 2:**
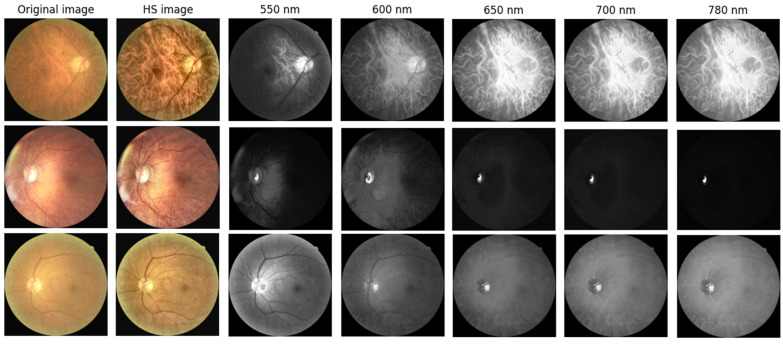
Representative Hyperspectral (HS) montages compared with original ophthalmoscopic images. The figure displays color-reconstructed HS images at specific spectral bands (550, 600, 650, 700, and 780 nm). These wavelength-dependent views reveal structural and vascular variations—such as vessel contrast and pigmentation—that are less discernible in standard RGB imaging. The range 550–780 nm was specifically selected to highlight these features.

**Figure 3:**
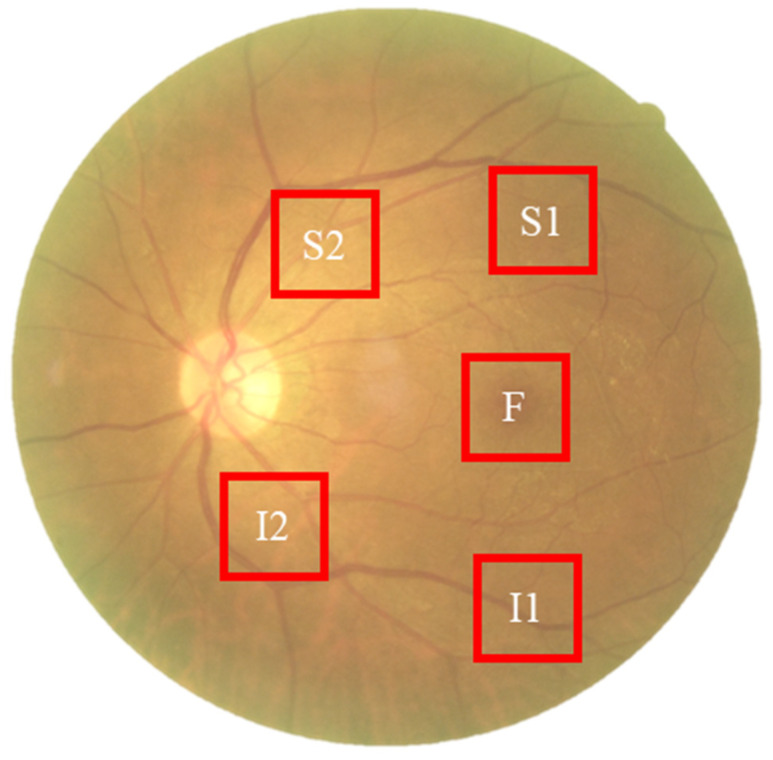
Schematic representation of the five Regions of Interest (ROIs) selected for spectral analysis. The standardized sampling areas (240 × 240 pixels) are located at: the fovea (F), the superior temporal arcade (S1, S2), and the inferior temporal arcade (I1, I2). These regions were chosen to ensure consistent spatial sampling of vascular and neural tissues across all participants.

To capture spectral variations associated with retinal pathology that are invisible to standard photography, we utilized a custom Hyperspectral Ophthalmoscope Imaging (HSOI) system. The system reconstructs high-fidelity spectral reflectance (380–780 nm) from standard RGB fundus images using a transformation matrix derived from a 24-color checkerboard calibration. Fundus images were captured using a Kowa Nonmyd 7 non-mydriatic retinal camera (Kowa Company Ltd., Nagoya, Japan). For the purpose of hyperspectral reconstruction calibration, we utilized an QE65000 spectrometer (Ocean Insight, Orlando, FL, USA). Detailed specifications of the optical setup, the spectrometer calibration process, and the mathematical formulation for spectral reconstruction are provided in the Supplementary Materials. Detailed mathematical formulations for the spectral reconstruction and color correction algorithms are provided in Supplementary Materials Section S1. Spectral Band Selection: From the full reconstructed cube (512 × 512 × 401), we specifically selected the 550–780 nm spectral range for analysis. This range was chosen to minimize short-wavelength scattering (Rayleigh scattering) and maximize penetration depth into the choroidal vasculature, where amyloid-related perfusion changes may occur. As shown in Figure 2, this band selection reveals distinct retinal features—including vasculature and pigmentation variations—that differ from standard RGB representations. ROI Extraction: Five standardized Regions of Interest (ROIs) were extracted from each image: the superior temporal arcade (S1, S2), the fovea (F), and the inferior temporal arcade (I1, I2), as illustrated in Figure 3. These regions were strategically selected based on physiological relevance:

Temporal Arcades (S1, S2, I1, I2): These areas have high nerve fiber layer thickness and vascular density, making them sensitive to potential amyloid deposition and microvascular alterations. Fovea (F): This region reflects macular pigment density and photoreceptor integrity, which correlate with central nervous system health. Dataset Preparation: To evaluate the added value of spectral data, two parallel datasets were constructed for deep learning training: (1) Original Retinal Images (ORIs) and (2) Reconstructed Hyperspectral Images (HSIs). This comparative design allows for the specific assessment of whether hyperspectral features improve diagnostic accuracy over conventional imaging.

The collected ophthalmoscopic images were sampled in specific areas. The regions are selected to ensure correlation between spectral variables and to avoid selection bias. Sampling areas with high color contrast representation because of the presence of vascular organizations, biological organs of the eye such as fovea, nerve fiber, which helps to enhance the spectra bands [19]. The sampling locations were set in five positions: above the temporal vascular arcade (S1 and S2), the fovea (F), and below the temporal vascular arcade (I1 and I2). The area size was 240 × 240 pixels (Figure 3). The images were then transformed into 401 bands of visible-light spectrum information using hyperspectral conversion technology. HS imaging algorithm for ophthalmoscopic images with mathematical derivation, validation of this reconstruction algorithm, and its accuracy metrics have been extensively described in our previous studies [48,49,50] and are summarized in Supplementary Materials Section S1. The resulting spectral information contained 401 spectral channels within the visible-light band (380 nm to 780 nm). The five positions, S1, S2, F, I1, and I2, are obtained as hyperspectral signals. Data preprocessing was performed to standardize the input for the deep learning models while strictly preserving the physiological fidelity of the spectral data. The procedures included: Spatial Resizing: Images were resized to [240 × 240] pixels to match the network input dimensions. This operation utilizes bicubic interpolation, which affects spatial resolution but does not alter the spectral value of individual pixels. Intensity Normalization: Pixel values were normalized to the range [0, 1]. This is a linear transformation that scales the data for optimal model convergence without distorting the relative ratios between spectral bands. Crucially, no non-linear contrast enhancement (e.g., histogram equalization) was applied to the hyperspectral data, ensuring that the spectral curves reflecting tissue properties (e.g., hemoglobin absorption, pigmentation) remained intact for valid physiological interpretation. The stages of dementia, including normal, MCI, and dementia, are detected by analyzing the differences in spectral intensity on the diagram.

### 2.3. Training with Deep Learning Models

To prevent data leakage and ensure unbiased evaluation, the dataset partitioning was conducted at the patient level. Specifically, all images from a single participant were assigned entirely to either the training or the testing set. This ensures that the model is evaluated on images from individuals it has never seen during training. To enhance dataset variability and improve model generalization, a comprehensive set of preprocessing techniques was applied. These included green channel extraction to highlight retinal vascular features, controlled pixel-level noise augmentation to improve robustness against imaging artifacts, morphological image opening for noise reduction, and spatial transformations such as rotation and horizontal flipping to expand spatial diversity within the training data. Through these preprocessing steps, the dataset was effectively augmented, producing a wider range of variations and enhancing model stability during both training and evaluation. Deep learning experiments were conducted using the PyTorch (version 1.12.1) framework with transfer learning to optimize classification accuracy across four neural network architectures. The training process employed a cross-entropy loss function, with the loss progressively minimized after each epoch to refine model weights. The batch size was set to 16, and training was performed for 50 epochs with an initial learning rate of 0.001. To facilitate stable convergence, the learning rate was reduced to one-tenth of its previous value every seven epochs. All CNN architectures were implemented using the TensorFlow/Keras framework. The specific training hyperparameters were set as follows: Input Resolution: Resized to 224 × 224 pixels for ResNet50, GoogLeNet, and EfficientNet; and 299 × 299 pixels for Inception_v3. Optimizer: Adam optimizer (Adaptive Moment Estimation). Learning Rate: Initial learning rate set to 0.0001 (1 × 10^−4^). Batch Size: 32. Epochs: 50 epochs. Loss Function: Categorical Cross-Entropy. Hardware: Training was performed on an NVIDIA GeForce RTX 3090 GPU.

### 2.4. Data Partitioning and Validation Strategy

#### 2.4.1. CNN Architectures

To evaluate the diagnostic potential of HSI versus ORI, we implemented four state-of-the-art Convolutional Neural Network (CNN) architectures: ResNet50, Inception_v3, GoogLeNet, and EfficientNet-B0. These models were selected for their proven performance in medical image classification. We utilized transfer learning by initializing the models with weights pre-trained on the ImageNet dataset. The final fully connected layers of each network were replaced with a custom classification head tailored for our binary task (Dementia vs. Normal), consisting of a global average pooling layer, a dropout layer (rate = 0.5) to mitigate overfitting, and a final dense layer with softmax activation.

#### 2.4.2. Data Partitioning and Cross-Validation

To ensure rigorous evaluation and prevent data leakage, data partitioning was performed strictly at the patient level. This ensures that all images (ROIs) belonging to the same subject were assigned exclusively to either the training, validation, or testing set. In each fold, the dataset was randomly divided into training (70%), validation (10%), and testing (20%) subsets. This approach guarantees that the model is tested on unseen subjects, providing a realistic estimate of clinical generalization.

#### 2.4.3. Training Configuration

The models were implemented using the PyTorch framework on an NVIDIA GeForce RTX 3090 GPU. Image preprocessing included resizing to 240 × 240 pixels and normalization to the [0, 1] range. To enhance model robustness and prevent overfitting, online data augmentation was applied to the training set, including random horizontal/vertical flips and random rotations (±15 degrees).

Training was conducted using the Adam optimizer with an initial learning rate of 1 × 10^−4^ and a batch size of 32. We used the Binary Cross-Entropy (BCE) loss function. The training process ran for a maximum of 50 epochs, with an early stopping mechanism (patience = 10 epochs) monitoring the validation loss to terminate training when no improvement was observed.

#### 2.4.4. Statistical Analysis

Model performance was evaluated using Accuracy, Sensitivity, Specificity, and the Area Under the Receiver Operating Characteristic Curve (AUC). We reported the mean and standard deviation across the 5 folds. To statistically compare the performance differences between HSI-based and ORI-based models, a paired *t*-test was performed, with a *p*-value < 0.05 considered statistically significant.

## 3. Results

To further examine the influence of age and disease on retinal spectral characteristics, comparative analyses were conducted to investigate age-dependent and disease-specific variations.

**Figure 4:**
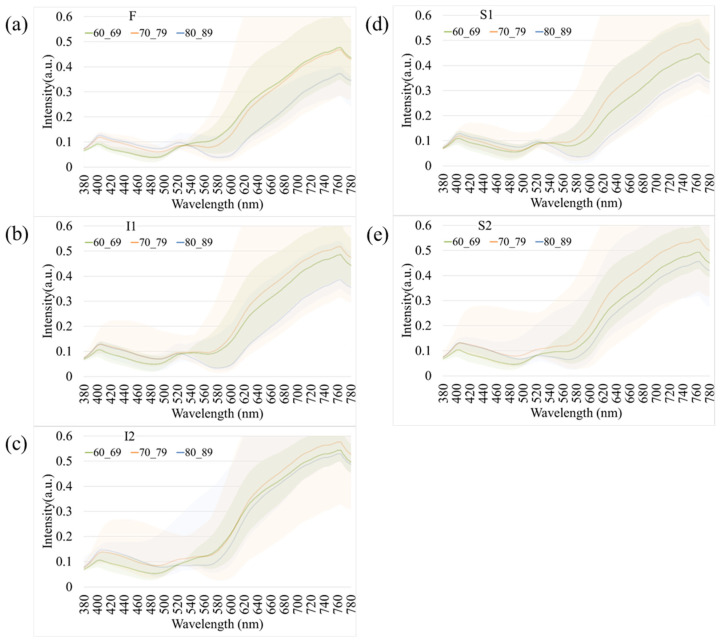
Age-dependent variations in retinal spectral reflectance among cognitively normal participants. The panels display mean spectral reflectance curves for three age groups (60 s, 70 s, and 80 s) across five retinal regions: (**a**) Fovea (F), (**b**) Inferior 1 (I1), (**c**) Inferior 2 (I2), (**d**) Superior 1 (S1), and (**e**) Superior 2 (S2). Shaded bands represent the spectral variability (range). A progressive increase in reflectance intensity is observed in the longer wavelength range (>600 nm) for the oldest age group (80 s), particularly in the S1 region.

Figure 4 presents age-related spectral variations derived from normal retinal images. Across all five retinal regions (F, I1, I2, S1, and S2), the reflectance spectra of participants in their 60 s, 70 s, and 80 s exhibited substantial overlap in the short-wavelength range (380–530 nm), indicating minimal age-dependent optical differences. In contrast, a progressive increase in reflectance was observed at longer wavelengths (>530 nm), particularly among individuals aged 80 years and above. This trend was most evident in the S1 region, suggesting that the superior temporal retina is more sensitive to age-associated optical and structural alterations. The shaded bands in Figure 4 denote the range of spectral variability across age groups, confirming that the most pronounced divergence occurred within the 600–780 nm wavelength range. These findings imply that aging predominantly affects retinal reflectance in the red to near-infrared spectrum, potentially due to cumulative changes in retinal tissue composition, vascular density, and pigment distribution.

**Figure 5:**
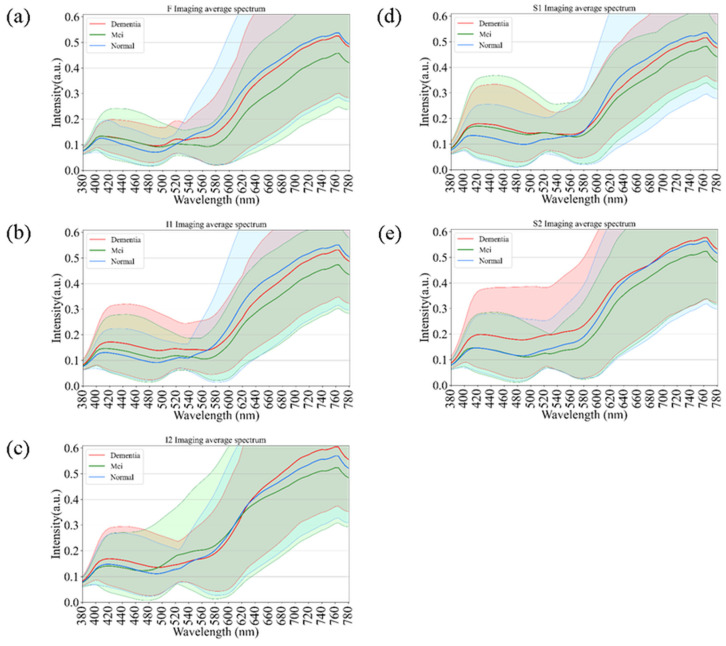
Comparative spectral reflectance profiles distinguishing Normal, Mild Cognitive Impairment (MCI), and Dementia groups. The graphs illustrate the mean spectral intensity across five retinal regions: (**a**) F, (**b**) I1, (**c**) I2, (**d**) S1, and (**e**) S2. Solid lines represent the mean reflectance, while shaded areas indicate the standard deviation. A statistically significant increase in reflectance is evident in the long-wavelength range (600–780 nm) for the Dementia group compared to the Normal and MCI groups, particularly in regions S1 and I2.

Figure 5 illustrates the comparative spectral reflectance profiles of retinal regions across different stages of cognitive impairment, including normal cognition, mild cognitive impairment (MCI), and dementia. Across all five analyzed regions (F, I1, I2, S1, and S2), the spectral curves followed a broadly similar pattern, with negligible divergence in the short-wavelength range (380–550 nm). Figure 5 illustrates the average spectral reflectance curves for the Dementia and Normal groups. The solid lines represent the mean reflectance, while the shaded areas indicate the standard deviation (SD), reflecting the variability within each group. Crucially, despite the known influence of aging on retinal reflectance (as seen in Figure 4), the Dementia group exhibited a statistically significant increase in reflectance intensity compared to the Normal group, particularly in the long-wavelength range (600–700 nm). A two-tailed *t*-test confirmed that these spectral differences were statistically significant (*p* < 0.05) across the highlighted bands. This suggests that the observed spectral alteration is likely driven by pathological changes associated with dementia (e.g., amyloid deposition or microvascular alterations) rather than demographic factors alone. In contrast, a progressive elevation in reflectance intensity was observed at longer wavelengths (>550 nm) as cognitive function declined from normal to MCI and then to dementia. This wavelength-dependent increase was most pronounced in the S1 and I2 regions, suggesting that hyperspectral reflectance within these retinal zones may serve as potential optical markers of neurodegenerative progression. The shaded areas in Figure 5 indicate the standard deviation across subjects within each diagnostic category, showing partial overlap among groups. Nevertheless, dementia cases consistently exhibited slightly higher reflectance levels, particularly in the red to near-infrared region (650–780 nm). These results support the potential of hyperspectral imaging to identify early cognitive impairment through subtle, wavelength-specific alterations in retinal reflectance.

**Figure 6:**
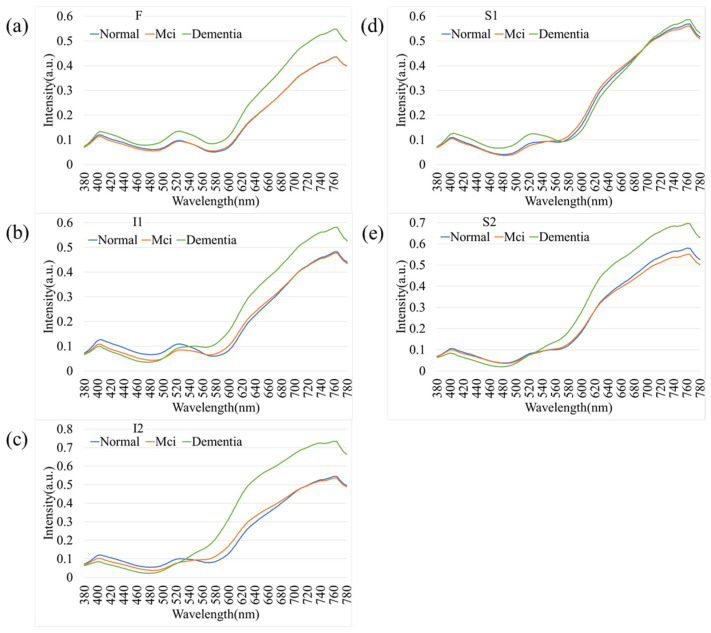
Spectral reflectance profiles of female participants stratified by cognitive status. Comparison of Normal, MCI, and Dementia groups across five retinal regions: (**a**) F, (**b**) I1, (**c**) I2, (**d**) S1, and (**e**) S2. Similarly to the overall population, female participants with dementia exhibit higher spectral reflectance in the longer wavelengths (>650 nm), with the most distinct separation observed in the inferior (I2) and superior (S2) temporal arcades.

As an extension of the overall spectral analysis, Figure 6 depicts the comparative spectral reflectance profiles of female participants across different stages of cognitive decline—normal cognition, mild cognitive impairment (MCI), and dementia. Across all five retinal regions (F, I1, I2, S1, and S2), the spectral curves exhibited a consistent overall pattern, with minimal separation among groups in the short-wavelength range (380–550 nm). In contrast, a pronounced increase in reflectance was observed at longer wavelengths (>550 nm), particularly beyond 650 nm, where dementia cases demonstrated higher spectral intensity compared with both MCI and normal participants. This wavelength-dependent enhancement was most evident in the I2 and S2 regions, suggesting that the inferior retinal zones may exhibit heightened sensitivity to neurodegenerative changes. The progressive elevation in reflectance from normal to MCI and dementia reflects the optical response of retinal tissues to disease advancement. These results highlight the potential of hyperspectral reflectance features within specific retinal areas as noninvasive optical biomarkers for detecting cognitive decline in female individuals.

**Figure 7:**
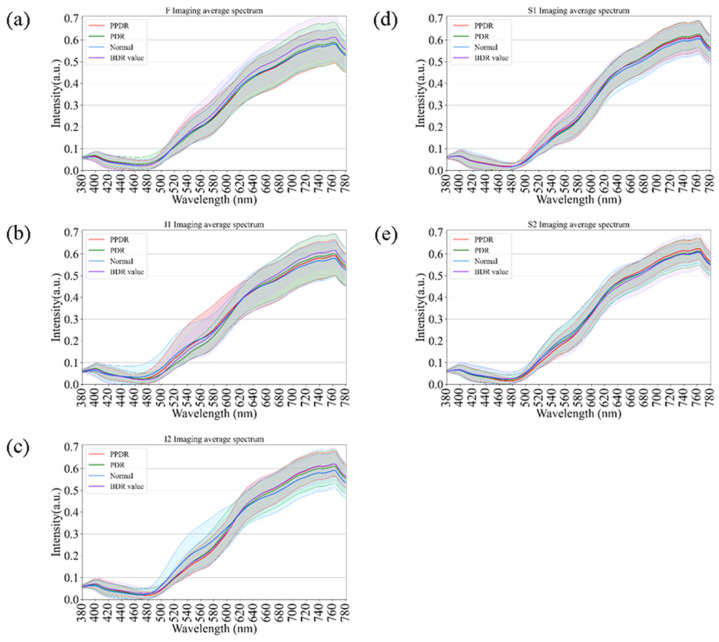
Comparison of retinal spectral variations across Diabetic Retinopathy (DR) severity stages. The curves show spectral reflectance for Normal controls, Background DR (BDR), Pre-proliferative DR (PPDR), and Proliferative DR (PDR) across regions (**a**) F, (**b**) I1, (**c**) I2, (**d**) S1, and (**e**) S2. A progressive elevation in long-wavelength reflectance correlates with increasing disease severity, serving as a comparative reference for spectral changes driven by retinal pathology.

As an extension of the spectral analysis, Figure 7 illustrates the comparative spectral reflectance profiles of diabetic retinas across different disease stages, including background diabetic retinopathy (BDR), pre-proliferative diabetic retinopathy (PPDR), proliferative diabetic retinopathy (PDR), and normal controls. Across all retinal regions (F, I1, I2, S1, and S2), the spectral curves displayed consistent overall trends, with only minor variations in the short-wavelength range (380–550 nm). In contrast, at longer wavelengths (>550 nm), a progressive elevation in reflectance was observed with increasing disease severity, from BDR to PPDR and PDR. This enhancement was particularly pronounced in the I2 and S1 regions, suggesting that progressive microvascular damage and retinal structural remodeling may alter optical scattering characteristics in these areas. Although partial spectral overlap was evident among stages, hyperspectral analysis revealed wavelength-dependent reflectance differences associated with disease progression. These findings support the potential utility of hyperspectral reflectance as a complementary, noninvasive indicator for evaluating the severity and progression of diabetic retinopathy.

**Figure 8:**
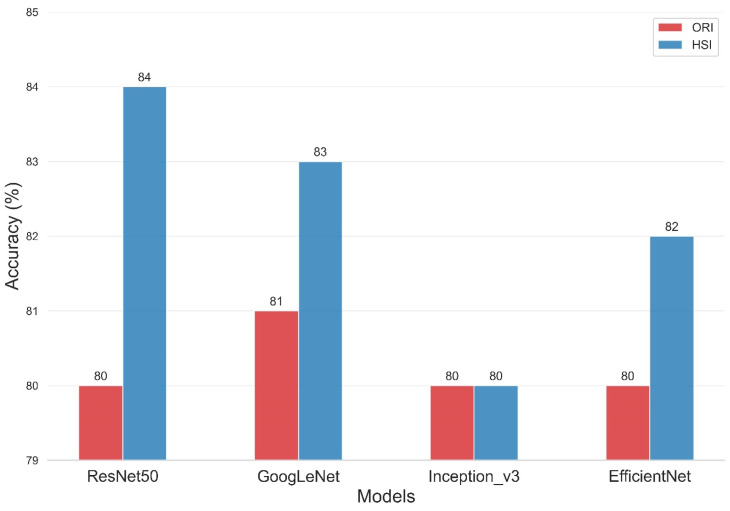
Classification accuracy comparison of four Deep Learning models on Original Retinal Images (ORI) versus Hyperspectral Images (HSI). The bar chart displays the overall accuracy achieved by ResNet50, GoogLeNet, Inception_v3, and EfficientNet. HSI-based models (orange bars) consistently outperform ORI-based models (blue bars), with ResNet50 achieving the highest accuracy of 84% using hyperspectral data.

The present study compared the evaluation of four deep-learning models using two types of data sets, namely, ORIs and HSIs, for dementia diagnosis. Figure 8 and Table 2 illustrate the improvement in the accuracy of the models upon incorporating HSIs into their evaluation. The overall accuracy of the ResNet50 training model was 80% for the ORI and 84% for the HSI. Similarly, the Inception_v3 model had an overall accuracy of 80% for both ORI and HSI, whereas the GoogLeNet model showed overall accuracies of 81% for ORI and 83% for HSI. The overall accuracies of the EfficientNet model reached 80% for ORI and 82% for HSI. The accuracies of ResNet50, GoogLeNet, and EfficientNet increased by 4% (from 80% in ORIs to 84% in HSIs), 2% (from 81% in ORIs to 83% in HSIs), and 2% (from 80% in ORIs to 82% in HSIs), respectively. The accuracy of Inception_v3, however, remained constant from ORI (80%) to HSI (80%), but it still demonstrated an improvement in lesion detection capability. These results demonstrate that the use of HSIs, which provide spectral features, can improve accuracy compared with ORIs. This increase in accuracy can range from a minimum of 1% to a maximum of 4%, depending on the neural network model used. Moreover, these results indicate that the learning capability of different models varies with various image data, and thus, multiple models can be utilized for comparison in deep learning applications. Our results indicate that HSI-based models achieved an accuracy improvement of approximately 3–4% compared to ORI-based models (e.g., ResNet50: 84% vs. 80%). While this numerical increase may appear modest, it is statistically significant (*p* < 0.05) and clinically relevant. In early-stage dementia screening, even a marginal improvement in sensitivity can translate to a significant number of correctly identified cases, facilitating earlier intervention. However, we acknowledge that preprocessing steps and dataset size may influence these margins. Future studies with larger, multi-center cohorts are necessary to validate whether this performance gain is consistently maintained across diverse populations.

Table 2 presents the performance of various models on the ORI and HSI datasets. In the ORI dataset, the mean Precision of ResNet50, GoogLeNet, Inception_v3, and EfficientNet were 80%, 81%, 79%, and 80%, respectively. For the HSI dataset, the mean Precision of the same models were 83%, 83%, 79%, and 81%, respectively. The Precision values for each class were found to be quite high, with the MCI class demonstrating the highest precision in the HSI dataset. Specifically, ResNet50, GoogLeNet, Inception_v3, and EfficientNet achieved precision values of 86%, 86%, 85%, and 87%, respectively, for the MCI class in the HSI dataset. Similarly, the Specificity values were also found to be high for predicting the MCI class in the HSI dataset, with ResNet50, GoogLeNet, Inception_v3, and EfficientNet achieving Specificity values of 89%, 91%, 91%, and 92%, respectively. However, the Recall of MCI in ORI detection was found to be relatively low, with ResNet50, Inception_v3, GoogLeNet, and EfficientNet achieving recall values of 63%, 69%, 68%, and 65%, respectively. These results suggest that the MCI category is particularly challenging to detect. The detection capabilities of the four models in differentiating between dementia and normal conditions are significantly higher than those in the differentiation from MCI. This finding was due to MCI being a transitional phase of dementia. In addition, patients may not display noticeable differences in their daily lives but possibly experience significant memory decline. In HSI dataset, the Recall of the MCI class for ResNet50, Inception_v3, GoogLeNet, and EfficientNet were found to be 81%, 70%, 78%, and 71%, respectively, indicating a significant improvement in the capability to detect MCI through the incorporation of HSIs. The trade-off between Precision and Recall often results in models with high Precision and low Recall or low Precision and high Recall. Therefore, it is difficult to determine which model is better because it is unclear whether precision or recall should be prioritized in the evaluation. To address this issue, we introduced a new metric that combines both Precision and Recall, called the f1-score. The f1-score is the harmonic mean of Precision and Recall, and it provides a more balanced assessment of both measures. For cases where Precision and Recall differ significantly, the f1-score can help us make a more objective evaluation. Based on Table 2, the f1-score indicates that the improvement of models on the HSI dataset is superior to that on the ORI dataset. Specifically, the mean f1-score of ResNet50 on ORI is 80%, which increases to 83% on HSI. GoogLeNet’s f1-score also increases by 2%, from 81% on ORI to 83% on HSI. Similarly, the f1-score of EfficientNet increases by 2% from 79% on ORI to 81% on HSI. However, the f1-score for Inception_v3 remains at 79% and does not change. Although ROC analysis was not performed, the performance advantage of the HSI approach is evident in the class-wise evaluation metrics, particularly for the Mild Cognitive Impairment (MCI) class. Diagnosing MCI is often challenging due to its subtle pathological features. As shown in Table 2 (or derived from the confusion matrices), the ORI-based ResNet50 model achieved a Recall of only 63% for the MCI class. In contrast, the HSI-based model significantly boosted the MCI Recall to 81%, with a corresponding increase in the F1-score from 73% to 83%. A similar trend was observed in the GoogLeNet model (MCI Recall improved from 68% to 78%). This specific improvement demonstrates that the hyperspectral reconstruction successfully recovered latent spectral features critical for distinguishing early-stage cognitive decline, which were otherwise insufficient in the original RGB imagery.

## 4. Discussions

In our study, we observed a disparity in reflectance spectra primarily in the longer wavelength range (>550 nm), as depicted in Figure 4 and Figure 5. As the result, it is possible to detect different stages of dementia in male and female. Other factors, such as age, gender, and eye-related diseases, may affect the performance of the spectrum. Therefore, more research is required to establish a reliable foundation for spectral analysis of the influence of such factors on dementia. This discrepancy can be attributed to the distinct morphologies adopted by Aβ formations under varying conditions. The process of Aβ formation exerts an influence on the growth of fibrils, thereby contributing to the observed variations in reflectance.

The observed variations in longer wavelengths among the three different age groups (Figure 4) can be attributed to the differential presence of Aβ plaques and oligomers. The profiles shown in Figure 4 demonstrate an increase in the difference in reflectance at longer wavelengths. In a study conducted on 5xFAD mice, it was found that Aβ plaques increase with age. These findings suggest that light scatter is induced by the presence of oligomers in younger individuals. However, as we age, the oligomers diminish while Aβ plaques become more prevalent, potentially leading to enhanced scatter and a more pronounced effect at longer wavelengths. Our study reinforces the strong association between amyloid beta occurrence and light scattering, thereby contributing to the observed differences at longer wavelengths. This also aligns perfectly with the results from Hadoux et al. [19] when applying the spectral model to the validation dataset at sampling position S1, which is regarded as having the largest spectral difference. Previous studies have suggested that the accumulation of amyloid-beta (Aβ) plaques in the retina can alter light scattering properties, particularly in the longer wavelength spectrum [19]. However, it is important to note that this study did not perform direct histopathological confirmation of Aβ deposits. Therefore, while the observed spectral differences in the dementia group are consistent with the optical effects of macromolecular aggregation, they may also reflect other structural changes associated with neurodegeneration. Nevertheless, an important aspect of our study was the influence of the retinal vascular system, specifically the arteries and veins. With age, the concentration of lutein in the retina decreases, resulting in shrinkage of the arteries and veins [16,51,52,53,54]. This decrease in lutein concentration leads to a reduction in reflection intensity within the wavelength range of 580 nm to 780 nm, thus resulting in a significant difference in spectral intensity observed across various age groups.

We acknowledge that retinal spectral reflectance is influenced by physiological factors such as melanin density, hemoglobin absorption, and lens opacification. While our exclusion criteria removed cases with severe ocular pathology (e.g., AMD, Glaucoma), subtle age-related changes are inevitable. The use of mydriasis in our protocol helped standardize light entry and reduce pupil-dependent artifacts. However, obtaining high-quality peripheral retinal images in elderly patients with dementia remains challenging due to potential fixation difficulties. To mitigate this, our study focused on analyzing standardized Regions of Interest (ROIs) around the temporal arcades and fovea, ensuring that only clear, artifact-free regions were processed by the deep learning models.

A limitation observed in our study is the lower recall rate for the MCI class compared to Normal and Dementia classes. This is attributed to the subtle spectral changes in early-stage cognitive decline that are difficult to distinguish from healthy tissue. While standard data augmentation was employed, future work will specifically address this challenge by implementing class-weighted loss functions or Focal Loss to penalize misclassifications of the minority MCI class more heavily, thereby potentially improving the model’s sensitivity to early-stage disease markers. Thirdly, the spectral reconstruction process in this study relied on a standard 24-color X-Rite ColorChecker for calibration. It is important to acknowledge that the spectral reflectance profiles of inorganic pigments on the ColorChecker differ from the complex biological reflectance properties of human retinal tissues. Consequently, there may be discrepancies in the absolute accuracy of the reconstructed retinal spectra. Nevertheless, the relative spectral differences captured by our method proved sufficient to enhance the deep learning model’s performance, as evidenced by the improved classification accuracy in HSI-based models compared to standard RGB models. Future research aims to incorporate biologically relevant calibration targets or direct hyperspectral fundus measurements to further improve reconstruction fidelity. A critical aspect of our spectral reconstruction method (WiSARD) involves using PCA to extract basis functions from the X-Rite ColorChecker. We recognize that the spectral subspace defined by these inorganic color patches may not fully encapsulate the complex, non-linear spectral characteristics of biological retinal tissues (e.g., absorption by hemoglobin and melanin). Ideally, PCA should be performed on a dataset of real retinal spectra; however, obtaining such ground-truth data remains a significant challenge in the field. Despite this limitation, our empirical results suggest that the ColorChecker-derived basis functions are sufficiently robust to approximate the retinal spectra for classification purposes. The significant performance boost observed in HSI-trained models implies that the reconstructed data successfully captured spectral variations associated with neurodegeneration that are otherwise invisible in standard RGB images. Future work will focus on collecting in vivo hyperspectral retinal data to refine these basis functions.

Regarding the spectral reconstruction algorithm, we employed third-order polynomial terms to account for the non-linear response of the camera sensor. A potential concern with such high-order mappings on a small calibration set (24 patches) is the risk of overfitting. To mitigate this, we relied on the pseudo-inverse method for coefficient estimation, which provides a minimum-norm solution that tends to be more stable. Furthermore, the generalizability of the reconstruction model is implicitly validated by the downstream classification results. Since the retinal images represent ‘out-of-distribution’ data compared to the ColorChecker, an overfitted model would likely introduce noise that hampers classification. The fact that our HSI-based deep learning models achieved higher accuracy and recall than the baseline RGB models demonstrates that the reconstruction algorithm successfully generalized to biological tissue, extracting valid spectral features that aided in distinguishing between MCI, dementia, and normal controls. Quantitatively, our calibration method achieved an average ΔE_00_ of 4.07. In colorimetry, a ΔE_00_ value greater than 2.3 is generally considered perceptible to the human eye, indicating that the reconstructed images contain noticeable color deviations compared to the ground truth. Regarding the spectral reconstruction accuracy, we observed an average color difference ΔE_00_ of 4.07, with a maximum deviation of 9.40. It is important to note that the higher ΔE_00_ values were primarily associated with highly saturated artificial colors on the calibration target, which do not typically appear in biological retinal tissue. Within the relevant color gamut of the human retina, the reconstruction error remains low. Furthermore, the deep learning models employed in this study extract high-level spatial-spectral features and are generally robust to minor pixel-level variations.

However, the impact of this error on the downstream spectral analysis must be interpreted in the context of the application. For deep learning-based classification, absolute colorimetric accuracy is often less critical than the preservation of relative feature distinctiveness. As long as the reconstruction error is systematic and does not obscure the pathological features associated with Alzheimer’s disease, the CNN can still learn effective decision boundaries. Our experimental results support this, as the HSI-based models consistently outperformed the RGB baselines, demonstrating that the reconstructed spectral information provided discriminative value despite the imperfect color calibration.

Furthermore, we acknowledge that ocular media opacities, particularly cataracts, are prevalent in the elderly population and can affect fundus recording quality. Lens opacification can introduce light scattering and alter spectral reflectance properties. Although we implemented pharmacological mydriasis to standardize light entry and strictly excluded images with significant media opacity that prevented clear visualization of the fundus, mild lens opacification remains a potential confounding factor. While our analysis focused on the posterior pole (fovea and temporal arcades) where image clarity is generally better preserved than in the periphery, future studies could benefit from incorporating quantitative cataract grading or adaptive optics to further mitigate the impact of optical aberrations on hyperspectral reconstruction.

## 5. Conclusions

In this era of population aging, dementia and retinal diseases are traditionally viewed as conditions that arise due to aging. However, to date, their age of onset is decreasing, which implies the need to constantly innovate drug development and medical testing to provide better care for the detection and treatment of patients. The use of retinal imaging and AI has numerous applications, including the diagnosis and treatment of diseases such as glaucoma and diabetic retinopathy [49,55]. The integration of AI in medical imaging is an ongoing endeavor, and thus far, the predictive outcomes obtained through conventional RGB image data sources do not appear to produce significant advantages. This study demonstrated the potential of combining hyperspectral retinal imaging with deep learning algorithms (ResNet50, Inception_v3, GoogLeNet, and EfficientNet) to distinguish between normal controls, MCI, and dementia. Our results indicate that spectral reflectance differences, particularly in the long-wavelength region, are correlated with cognitive decline. However, we recognize that the current overall accuracy (approximately 80–84%) and the sensitivity for the MCI group are insufficient for immediate clinical deployment as a standalone diagnostic tool. The distinction between MCI and normal aging remains subtle, and the current model yields false negatives that must be addressed. Therefore, at this stage, this technology should be viewed as a non-invasive, cost-effective assistive screening tool, intended to identify high-risk individuals who require further comprehensive neurological evaluation.

To establish true diagnostic utility, the following concrete validation steps are essential for future research: External Validation: The models must be tested on independent datasets collected from different hospitals and using different fundus camera models to ensure generalizability and robustness against device-specific variations. Correlation with Gold Standards: Future studies should perform direct correlation analysis between retinal spectral features and established biomarkers, such as amyloid-PET scans, CSF analysis, or plasma biomarkers (e.g., p-Tau). This will provide the necessary biological validation that is currently missing. Longitudinal Studies: A longitudinal cohort study is required to track patients with MCI over time. This will allow us to determine if specific spectral changes can predict the conversion from MCI to dementia, thereby validating the prognostic value of retinal hyperspectral imaging. Multimodal Integration: To improve sensitivity for MCI, future models should integrate hyperspectral data with other modalities, such as OCT (Optical Coherence Tomography) structural data and clinical risk factors, to enhance diagnostic performance.

## Figures and Tables

**Table 1:** Demographic characteristics and dataset composition. The table details the distribution of participants (n = 137) and total images (n = 3256) across three cognitive groups: Normal Control, Mild Cognitive Impairment (MCI), and Dementia. Data includes gender breakdown, age ranges, and Mini-Mental State Examination (MMSE) score ranges.

	No. of Patients	Gender		Age	MMSE	No. of Images
Normal	49	Male	21	68~85	28~30	1254
		Female	28	60~82	27~30	
MCI	54	Male	19	61~82	23~27	1320
		Female	35	60~85	23~27	
Dementia	34	Male	12	68~87	12~23	682
		Female	22	60~80	13~22	

**Table 2:** Performance metrics of Deep Learning models on ORI vs. HSI datasets. The table presents the Accuracy, Precision, Recall, Specificity, and F1-score for ResNet50, GoogLeNet, Inception_v3, and EfficientNet. Results are stratified by dataset type (Original Retinal Images vs. Hyperspectral Images) and diagnostic class (Dementia, MCI, Normal). Note the improved Recall and F1-scores for the MCI class in the HSI-based models.

**ResNet50**					
**ORI**	Accuracy	Precision	Recall	Specificity	F1-score
Dementia	0.80	0.73	0.88	0.90	0.80
MCI		0.87	0.63	0.94	0.73
Normal		0.79	0.93	0.83	0.86
mean		0.80	0.82	0.89	0.80
**HSI**	Accuracy	Precision	Recall	Specificity	F1-score
Dementia	0.84	0.78	0.78	0.94	0.78
MCI		0.84	0.81	0.89	0.83
Normal		0.87	0.90	0.90	0.88
mean		0.83	0.83	0.91	0.83
**GoogLeNet**					
**ORI**	Accuracy	Precision	Recall	Specificity	F1-score
Dementia	0.81	0.78	0.81	0.93	0.79
MCI		0.88	0.68	0.93	0.77
Normal		0.78	0.96	0.81	0.86
mean		0.81	0.81	0.89	0.81
**HSI**	Accuracy	Precision	Recall	Specificity	F1-score
Dementia	0.83	0.79	0.79	0.94	0.79
MCI		0.86	0.78	0.91	0.82
Normal		0.83	0.91	0.87	0.87
mean		0.83	0.83	0.91	0.83
**Inception_v3**					
**ORI**	Accuracy	Precision	Recall	Specificity	F1-score
Dementia	0.80	0.69	0.77	0.90	0.73
MCI		0.86	0.69	0.92	0.76
Normal		0.82	0.94	0.85	0.88
mean		0.79	0.80	0.89	0.79
**HSI**	Accuracy	Precision	Recall	Specificity	F1-score
Dementia	0.80	0.67	0.83	0.88	0.74
MCI		0.85	0.70	0.91	0.77
Normal		0.84	0.89	0.88	0.87
mean		0.79	0.81	0.89	0.79
**EfficientNet**					
**ORI**	Accuracy	Precision	Recall	Specificity	F1-score
Dementia	0.80	0.73	0.81	0.91	0.77
MCI		0.89	0.65	0.94	0.75
Normal		0.78	0.96	0.81	0.86
mean		0.80	0.81	0.89	0.79
**HSI**	Accuracy	Precision	Recall	Specificity	F1-score
Dementia	0.82	0.71	0.88	0.89	0.78
MCI		0.87	0.71	0.92	0.78
Normal		0.85	0.89	0.88	0.87
Mean		0.81	0.83	0.90	0.81

## Data Availability

The data presented in this study are available in this article.

## Supplementary Materials

The following supporting information can be downloaded at: https://www.mdpi.com/article/10.3390/bioengineering12121362/s1, Figure S1: Schematic diagram of the proposed method, using an ophthalmoscope (funduscopy) to estimate the spectral reflectance of each pixel in an image. The Hyperspectral Ophthalmoscope Imaging Technology uses the standard 24 color blocks (X-Rite Classic, 24 Color Checkers) as the common target for the conversion of the ophthalmoscope and the spectrometer, and converts the ophthalmoscope image into 401 bands of visible spectrum information; Figure S2: XYZ color matching function; Figure S3: Polynomial regression graph of XYZ_Funducopy_ and XYZ_Spectrum_; Figure S4: Comparison of chromatic aberration between ophthalmoscope before and after correction and spectrometer; Figure S5: 12 principal components of R_Spectrum_; Figure S6: Root Mean Square Error of S_Spectrum_ and R_Spectrum_; Figure S7: Chromatic difference diagram of measured spectrum and simulated spectrum of 24 color blocks; Figure S8: shows the Loss curve and accuracy graph (ResNet50); Figure S9: Loss curve and accuracy plot (Inception_v3); Figure S10: Loss curve and accuracy plot (GoogLeNet); Figure S11: Loss curve and accuracy plot (EfficientNet); Table S1: Quantity of Imaging Data for Dementia; Table S2: Classification of Imaging Data in Dementia; Table S3: Dementia Assessment Criteria Results Table (ResNet50); Table S4: Dementia Assessment Criteria Results Table (Inception_v3); Table S5: Dementia Assessment Criteria Results Table (GoogLeNet); Table S6: Dementia Assessment Criteria Results Table (EfficientNet).
