# Supplementary material for: Dementia Detection via Retinal Hyperspectral Imaging and Deep Learning: Clinical Dataset Analysis and Comparative Evaluation of Multiple Architectures
Source: Bioengineering (Basel). 2025 Dec 14;12(12):1362. doi: 10.3390/bioengineering12121362 (PMC12730061; doi:10.3390/bioengineering12121362)
Supplement: Supplementary file 1 [file bioengineering-12-01362-s001.zip › bioengineering-4009826-supplementary.pdf]

bioengineering

Article

Dementia Detection via Retinal Hyperspectral Imaging and Deep Learning: Clinical Dataset Analysis and Comparative Evaluation of Multiple Architectures: Supplementary

Wen-Shou Lin [1,2], Chia-Ling Chen [3], Shih-Wun Liang [3] and Hsiang-Chen Wang [3,4,5,*]

1 Neurology Division, Department of Internal Medicine, Kaohsiung Armed Forces General Hospital, No. 2, Zhongzheng 1st Rd., Lingya District, Kaohsiung 80284, Taiwan; linvincent1009@gmail.com
2 Department of Medicine, National Defense Medical University, No. 161, Sec. 6, Minquan E. Rd., Neihu District, Taipei 11490, Taiwan
3 Department of Mechanical Engineering, National Chung Cheng University, 168, University Rd., Min Hsiung, Chiayi 62102, Taiwan; jolincchen@gmail.com (C.-L.C.); start90301@gmail.com (S.-W.L.)
4 Department of Medical Research, Dalin Tzu Chi Hospital, Buddhist Tzu Chi Medical Foundation, No. 2, Minsheng Road, Dalin, Chiayi 62247, Taiwan
5 Director of Technology Development, Hitspectra Intelligent Technology Co., Ltd., 4F, No. 2, Fuxing 4th Rd., Qianzhen District, Kaohsiung 80661, Taiwan
* Correspondence: hcwang@ccu.edu.tw

Academic Editors: Paul Constable, Hugo F. Posada-Quintero and Fabiano Bini

Received: 11 November 2025
Revised: 4 December 2025
Accepted: 10 December 2025
Published: 14 December 2025

Citation: Lin, W.-S.; Chen, C.-L.; Liang, S.-W.; Wang, H.-C. Dementia Detection via Retinal Hyperspectral Imaging and Deep Learning: Clinical Dataset Analysis and Comparative Evaluation of Multiple Architectures: Supplementary. *Bioengineering* **2025**, *12*, 1362. https://doi.org/10.3390/bioengineering12121362

Copyright: © 2025 by the author. Licensee MDPI, Basel, Switzerland. This article is an open access article distributed under the terms and conditions of the Creative Commons Attribution (CC BY) license (https://creativecommons.org/licenses/by/4.0/).

S1. Hyperspectral Ophthalmoscope Imaging Technology (HSOI Tech.)

The *HSOI Tech.* used in this research is to use the ophthalmoscope (Kowa Nonmyd 7, Torrance, CA, USA) and the spectrometer (Ocean Optics, QE65000, Dunedin, FL, USA) to find out the relationship between the two devices by shooting a common target. The process of *HSOI Tech.* was separated into three parts, which were spectral data reduction using PCA, calculation of the transformation matrix to determine the correlation between the ophthalmoscope and the spectrophotometer, and the spectral reproduction of images.

As the capture was corrected, the RGB values of each image element were processed to estimate the spectra accurately. Color correction was applied to match the color performance of the spectrophotometer and the camera. Standard RGB values were established using the International Commission on Illumination/Commission International de L'éclairage XYZ tristimulus values from 24-color checkers (Mini Color Checkers, X-Rite, Grand Rapids, MI, USA) based on spectrophotometric data. Under similar lighting conditions, images taken with endoscopy and a computer program were used to obtain the RGB values of each image element. Third-order polynomial regression was then performed separately for the RGB components to determine the color relationship between the two devices. As the xenon lamp in the output format of sRGB (RAW image files) in commercial endoscopy provided the reference white, which is different from the artificial lights used to measure the spectra of the 24-color checkers, chromatic adaptation transformation was carried out before third-order polynomial regression. The calculation procedure of the average spectra of ophthalmoscopic images are shown in Figure S1.

When conducting the modeling process, it is essential to ensure that both the spectral and image data collected are transformed into a consistent color gamut space, such as the CIE 1931 XYZ color space. The subsequent sections will provide a comprehensive explanation of the procedures involved in processing the data captured by the ophthalmoscope and spectrometer.

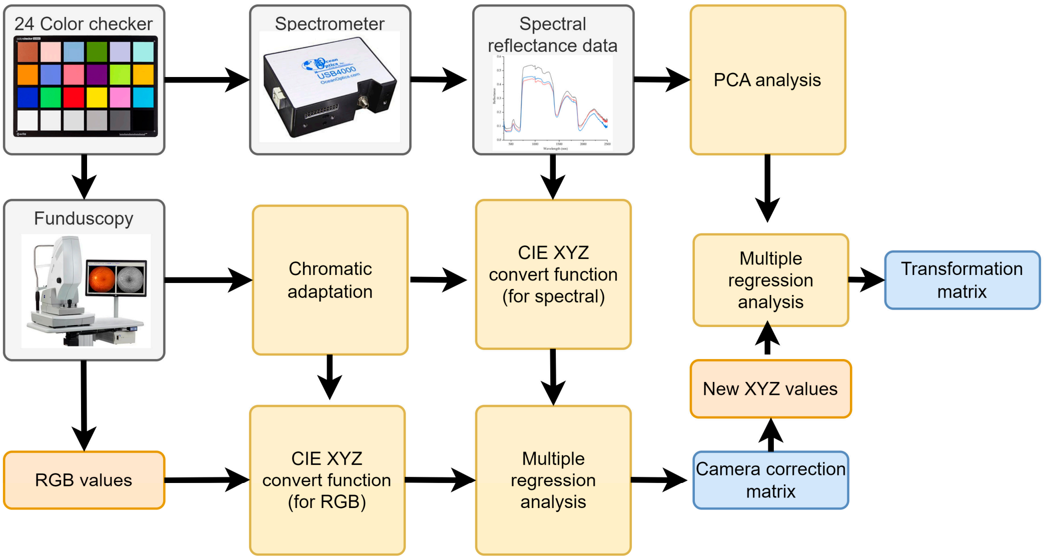

Figure S1. Schematic diagram of the proposed method, using an ophthalmoscope (funduscopy) to estimate the spectral reflectance of each pixel in an image. The Hyperspectral Ophthalmoscope Imaging Technology uses the standard 24 color blocks (X-Rite Classic, 24 Color Checkers) as the common target for the conversion of the ophthalmoscope and the spectrometer, and converts the ophthalmoscope image into 401 bands of visible spectrum information.

The images obtained through the ophthalmoscope are in the sRGB color gamut space, represented by JPEG, 24 Bit Depth, with pixel values ranging from 0 to 255. To achieve uniformity in the color representation, the conversion of these sRGB images to the XYZ color gamut space is performed using Equation 1, which is outlined as follows:

$$\begin{bmatrix} X \\ Y \\ Z \end{bmatrix} = [M_A][T] \begin{bmatrix} f(R_{sRGB}) \\ f(G_{sRGB}) \\ f(B_{sRGB}) \end{bmatrix} \times 100\,, 0 \leqq \begin{matrix} R_{sRGB} \\ G_{sRGB} \\ B_{sRGB} \end{matrix} \leqq 1, \quad (1)$$

From this equation, we can convert from sRGB to XYZ color gamut space and $XYZ_{funducopy}$ under the measurement light source.

Before converting to the XYZ color gamut space, it must be normalized to sRGB (0 ~ 1), and converted to linear RGB through the Gamma function, as shown in Equation 2.

$$f(n) = \begin{cases} \left(\frac{n+0.055}{1.055}\right)^{2.4}, & n > 0.04045 \\ \left(\frac{n}{12.92}\right), & otherwise \end{cases}, \quad (2)$$

Then convert the linear RGB to the XYZ color gamut space through the transformation matrix T, as shown in Equation 3.

$$[T] = \begin{bmatrix} 0.4104 & 0.3576 & 01805 \\ 0.2126 & 0.7152 & 0.0722 \\ 0.0193 & 0.1192 & 0.9505 \end{bmatrix}, \quad (3)$$

However, during the conversion, because the sRGB color gamut space defines the White point as the D65 light source (X_{CW}, Y_{CW}, Z_{CW}) rather than the white point of the measurement light source (X_{SW}, Y_{SW}, Z_{SW}), so we adapt the conversion matrix through the color M_A is used to convert the white point of the D65 light source into the white point of the measurement light source, as shown in Equation 4.

$$[M_A] = \begin{bmatrix} X_{SW}/X_{CW} & 0 & 0 \\ 0 & Y_{SW}/Y_{CW} & 0 \\ 0 & 0 & Z_{SW}/Z_{CW} \end{bmatrix}, \quad (4)$$

On the spectrometer side, converting the obtained spectral data R(λ) (380 nm ~ 780 nm, 1 nm) into the XYZ color gamut space requires the light source spectrum S(λ) when using the ophthalmoscope to shoot, and the XYZ color matching function (CMF) $\bar{x}(\lambda)$, $\bar{y}(\lambda)$, and $\bar{z}(\lambda)$, as shown in Figure S3, since the Y value of the XYZ color gamut space is proportional to the brightness, the Y value of the light source spectrum (maximum brightness), and the upper limit of the Y value is set to 100 to obtain the brightness ratio k, as shown in Equation 5.

$$k = 100/\int_{380nm}^{780nm} S(\lambda)\bar{y}(\lambda)\ d\lambda, \quad (5)$$

Finally, through equations 6, 7, and 8, the spectral data is converted into the XYZ value ($XYZ_{spectrum}$) regulated in the XYZ color gamut space.

$$X = k\int_{380nm}^{780nm} S(\lambda)R(\lambda)\bar{x}(\lambda)\ d\lambda, \quad (6)$$

$$Y = k\int_{380nm}^{780nm} S(\lambda)R(\lambda)\bar{y}(\lambda)\ d\lambda, \quad (7)$$

$$Z = k\int_{380nm}^{780nm} S(\lambda)R(\lambda)\bar{z}(\lambda)\ d\lambda, \quad (8)$$

After obtaining $XYZ_{funducopy}$ and $XYZ_{spectrum}$, it is essential to account for potential error factors during ophthalmoscope shooting, such as nonlinear response, dark current, and color shift. To address these issues, a correction matrix C is derived through multiple regression analysis (Equation 9). The correction matrix C allows for precise calibration of the ophthalmoscope, ensuring more accurate and reliable data for subsequent analysis and modeling.

$$[C] = [XYZ_{spectrum}] \times pinv([V]), \quad (9)$$

In the analysis, the spectrometer exhibits a linear response. However, when comparing the Y values (brightness) of the 19th to 24th color blocks from both the 24 color checker taken by the ophthalmoscope and the spectrometer, and performing linear regression analysis on the Y values of $XYZ_{funduscopy}$ and $XYZ_{spectrum}$ as shown in Figure S4, it becomes evident that the ophthalmoscope shows a nonlinear response. The coefficient of determination R2 in the third-order linear regression reaches 0.9725, indicating a strong similarity between the ophthalmoscope's nonlinear response and the third-order equation. Therefore, it is inferred that the ophthalmoscope's nonlinear response can be corrected using a third-order equation, and the correction variable is denoted as $V_{non\text{-}linear}$ (Equation 10). This correction will enhance the accuracy of the ophthalmoscope's data and improve the reliability of subsequent analyses and modeling.

$$V_{non-linear} = [X^3\ Y^3\ Z^3\ X^2\ Y^2\ Z^2\ X\ Y\ Z\ 1]^T, \quad (10)$$

Regarding the dark current, it remains constant and does not vary with the amount of light. Therefore, it is typically represented as a fixed value, and we define the dark current correction variable as V_{dark} (Equation 11). This correction factor accounts for the influence of dark current, allowing us to compensate for this constant component and ensure more accurate data from the ophthalmoscope. The inclusion

of V_{dark} in the correction process further enhances the reliability of the measurements and analysis.

$$V_{dark} = [\alpha], \tag{11}$$

In the color shift part, there is a problem with color matching. Since the ophthalmoscope image has been converted into the XYZ color gamut space, the relationship between X, Y, and Z values needs to be considered. As shown in the XYZ color matching function in Figure S3, $\bar{x}$, $\bar{y}$, and $\bar{z}$ are related in spectral distribution, so all possibilities among X, Y, and Z are listed and defined as V_{color}, as shown in Equation 12.

$$V_{color} = [XYZ\ XY\ YZ\ XZ\ X\ Y\ Z]^T, \tag{12}$$

After considering all the errors, we use V_{color} as the base to multiply $V_{non\text{-}linear}$ for nonlinear response correction. In order to avoid over-correction, the result is standardized in the third order, and finally, V_{dark} is added to obtain the variable matrix V, as shown in Equation 13, and finally, we bring the obtained variable matrix V back to Equation 9 to obtain the correction matrix C.

$$V = [X^3\ Y^3\ Z^3\ X^2Y\ X^2Z\ Y^2Z\ XY^2\ XZ^2\ YZ^2 \\ XYZ\ X^2\ Y^2\ Z^2\ XY\ XZ\ YZ\ X\ Y\ Z\ \alpha]^T, \tag{13}$$

Through Equation 9, we extend $XYZ_{funduscopy}$ to V matrix to obtain corrected X, Y, and Z values ($XYZ_{correct}$), as shown in Equation 14.

$$[XYZ_{correct}] = [C] \times [V], \tag{14}$$

As the wavelength band used in this study falls within the visible light spectrum (380 nm ~ 780 nm), the correction result can be expressed in terms of chromatic aberration. To calculate chromatic aberration, the study adopts the CIEDE 2000 method, which incorporates various techniques such as hue rotation, neutral color compensation, brightness compensation, chromaticity compensation, and tone compensation. The CIEDE 2000 method is employed to account for the differing sensitivity of human eyes to various colors. By utilizing these techniques, the correction process aims to minimize discrepancies in color perception and ensure a more accurate representation of color across the entire visible light spectrum. This approach enhances the overall reliability and consistency of the data, allowing for more robust analyses and interpretations.

Before applying the CIEDE 2000 method, it is necessary to convert the $XYZ_{correct}$ and $XYZ_{spectrum}$ data to the Lab color gamut space using equations 15, 16, 17, and 18:

$$L^* = 116 f\left(\frac{Y}{Y_n}\right) - 16, \tag{15}$$

$$a^* = 500\left[f\left(\frac{X}{X_n}\right) - f\left(\frac{Y}{Y_n}\right)\right], \tag{16}$$

$$b^* = 500\left[f\left(\frac{X}{X_n}\right) - f\left(\frac{Y}{Y_n}\right)\right], \tag{17}$$

$$f(n) = \begin{cases} n^{\frac{1}{3}} &, n > 0.008856 \\ 7.787n + 0.137931 &, otherwise \end{cases}, \tag{18}$$

Based on the results obtained from the CIEDE 2000 chromatic aberration calculation, significant chromatic aberration is observed between the ophthalmoscope and the spectrometer before correction. The average color difference between the two reaches 21.4, indicating a substantial deviation in color representation. After

implementing the correction process, the average color difference between the ophthalmoscope and the spectrometer decreases significantly to 4.07. This substantial reduction in color difference signifies a successful correction of the chromatic aberration issue. The visual representation in Figure S5 further confirms the effectiveness of the correction. Visually, the color difference between the two instruments is noticeably reduced after the correction, resulting in more consistent and accurate color representation across the visible light spectrum.

Principal component analysis (PCA) was conducted on $R_{spectrum}$, and it was observed that the first 12 sets of principal components (EV) can fully express 99.99% of the data variation, as depicted in Figure S6. Therefore, for dimensionality reduction, these 12 sets of principal components were utilized to obtain principal component scores. Subsequently, multivariate regression analysis was performed based on this reduced data.

V_{color} was included as a variable due to its comprehensive listing of correlations between X, Y, and Z. By incorporating V_{color}, a transformation matrix M was derived between the ophthalmoscope and the spectrometer, as described in Equation 19.

With the obtained transformation matrix, spectral conversion was performed on $XYZ_{correct}$, resulting in the analog spectral value $S_{spectrum}$ (Equation 20). This conversion process accounts for the discrepancies between the ophthalmoscope and spectrometer, ensuring a more accurate and consistent representation of spectral data.

$$[M] = [Score] \times pinv([V_{color}]), \tag{19}$$

$$[S_{spectrum}]_{380-780nm} = [EV][M][V_{color}], \tag{20}$$

After obtaining the simulated spectrum, a comparison is made with the reflection spectrum ($R_{spectrum}$) of the 24-color card. By calculating the Root-mean-square error (RMSE) between the two, the average RMSE is found to be 0.080, indicating a relatively small difference between the simulated and actual spectra.

To further assess the color difference, the simulated spectrum is converted to the Lab color gamut space for CIEDE 2000 color difference comparison. The average color difference is calculated to be 4.07, as shown in Figure S8. This value signifies the degree of chromatic aberration between the simulated spectrum and the 24-color checker spectrum after correction. The results demonstrate that the correction process has significantly reduced the discrepancies between the simulated and actual spectra. The average RMSE and color difference values indicate a notable improvement in color accuracy, validating the effectiveness of the correction method in achieving more reliable and consistent spectral data.

Color reproduction aims to map a spectrum of wavelengths to a corresponding color representation. In this study, the formulation relies on the CIE color matching functions $\bar{x}(\lambda)$, $\bar{y}(\lambda)$, and $\bar{z}(\lambda)$. By employing Equations 5, 6, 7, and 8, X, Y, and Z values are obtained from the spectral signal within the wavelength range of 550 nm to 700 nm. Once the XYZ color gamut space has been determined, the final step involves converting it to the sRGB color space. This conversion is achieved through the Gamma function, as specified in Equation 1. The Gamma function ensures a consistent and accurate transformation of the XYZ color data into the sRGB color representation, allowing for faithful color reproduction across different devices and platforms.

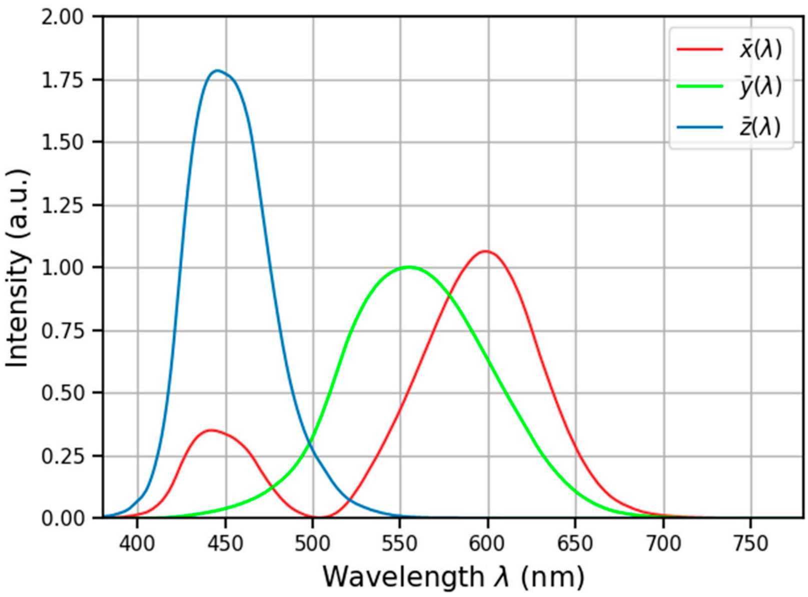

Figure S2. XYZ color matching function.

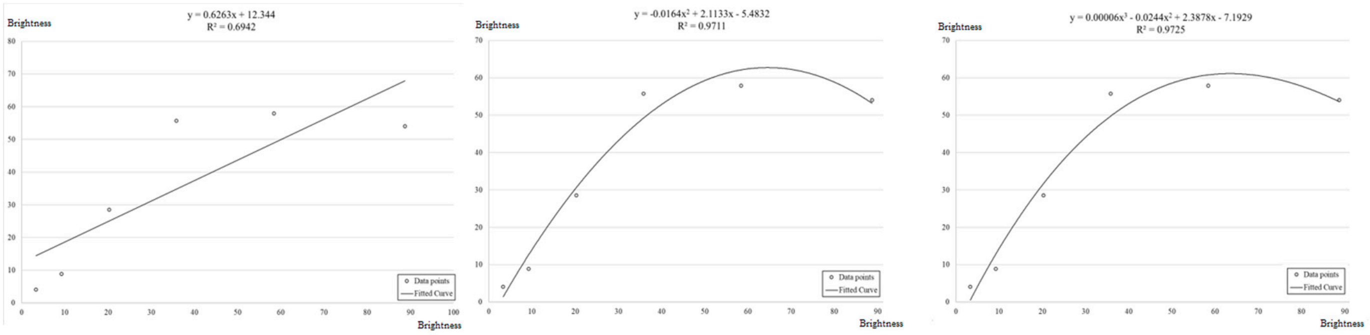

Figure S3. Polynomial regression graph of $XYZ_{Funducopy}$ and $XYZ_{Spectrum}$.

Ophthalmoscope measurement						
Color block No.	before correction	spectrometer	color difference	after correction	spectrometer	color difference
1			31.37			7.71
2			15.55			5.37
3			31.77			2.85
4			28.81			1.65
5			25.70			2.79
6			21.13			2.41
7			11.55			4.74
8			32.17			5.52
9			19.28			1.44
10			20.24			8.16
11			14.78			1.41
12			14.20			2.85
13			15.34			4.08
14			27.55			1.21
15			20.44			2.89
16			9.09			3.57
17			20.23			1.19
18			36.45			1.60
19			12.33			9.41
20			14.47			3.39
21			22.67			7.12
22			27.03			2.50
23			25.05			7.89
24			16.33			5.98
		Average	21.40		Average	4.07

Figure S4. Comparison of chromatic aberration between ophthalmoscope before and after correction and spectrometer.

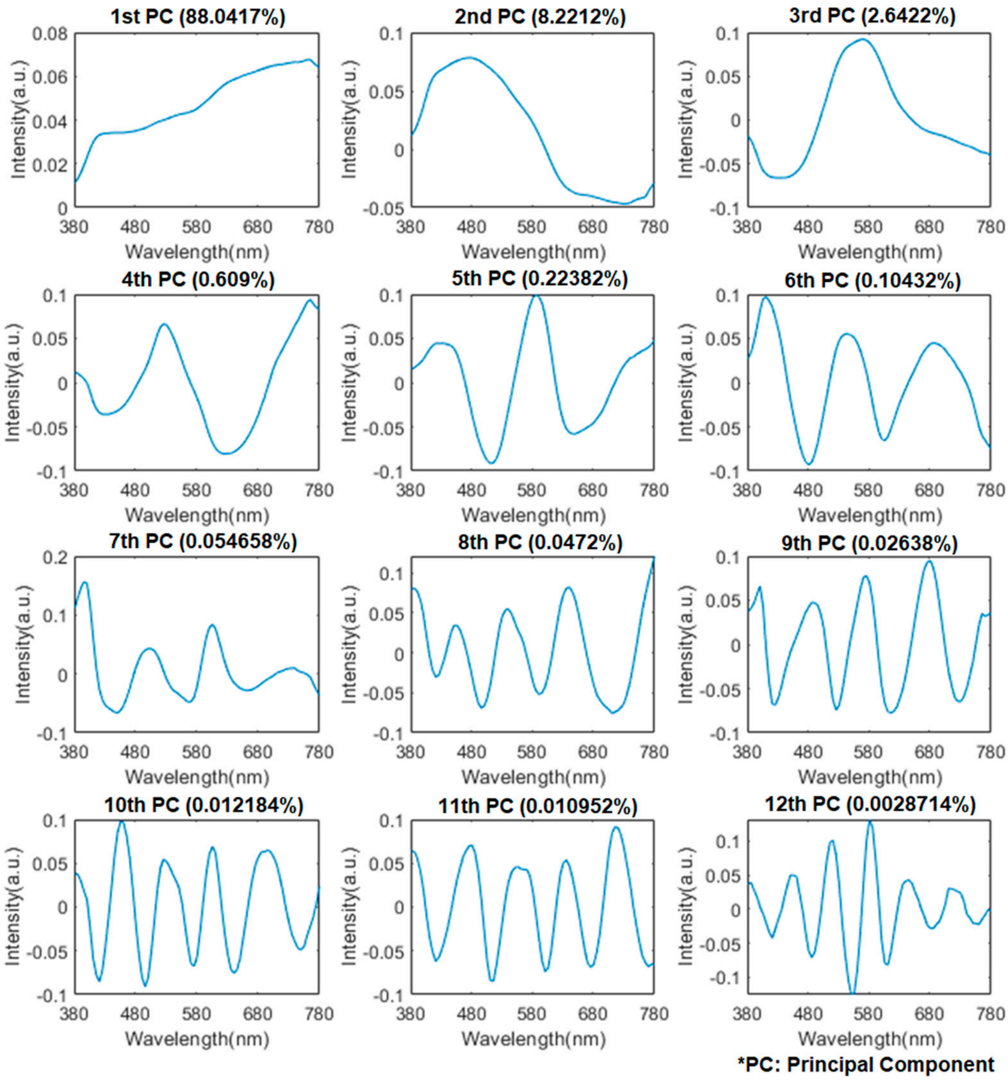

Figure S5. 12 principal components of $R_{Spectrum}$.

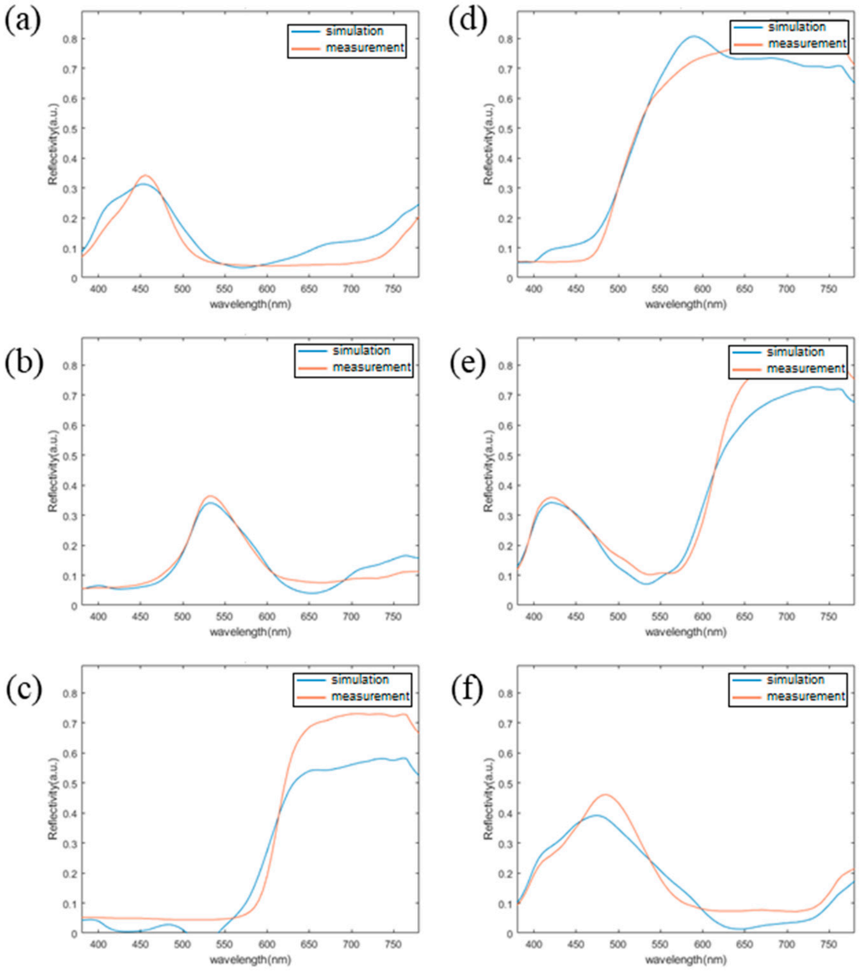

Color No.	corresponding graph	RMSE
1	-	0.051
2	-	0.067
3	-	0.112
4	-	0.096
5	-	0.091
6	-	0.042
7	-	0.060
8	-	0.039
9	-	0.065
10	-	0.144
11	-	0.014
12	-	0.048
13	(a)	0.045
14	(b)	0.026
15	(c)	0.087
16	(d)	0.058
17	(e)	0.062
18	(f)	0.051
19	-	0.335
20	-	0.034
21	-	0.207
22	-	0.124
23	-	0.033
24	-	0.026
Average error		0.080

Figure S6. Root Mean Square Error of $S_{Spectrum}$ and $R_{Spectrum}$.

Color blocks	measured spectrum			color display	simulated spectrum			color display	color difference
	L*	a*	b*		L*	a*	b*		
1	45.016	36.80	33.01		38.683	24.80	25.98		7.71
2	70.915	38.36	29.88		67.693	32.12	33.71		5.39
3	52.567	5.83	-9.17		49.764	5.64	-8.36		2.85
4	42.819	-6.15	28.46		42.363	-4.54	30.12		1.65
5	54.380	18.57	-9.82		56.213	21.88	-8.79		2.78
6	70.376	-14.16	13.97		69.557	-17.74	14.96		2.39
7	59.025	47.05	69.79		64.437	45.86	70.93		4.73
8	34.505	25.42	-33.42		39.658	18.18	-28.63		5.52
9	54.822	55.34	31.88		54.577	59.58	32.07		1.44
10	26.276	25.15	-20.66		31.387	30.57	-10.24		8.14
11	69.960	-9.07	67.76		71.731	-8.41	67.26		1.41
12	75.208	30.22	84.32		74.084	33.13	78.99		2.86
13	32.210	22.74	-33.58		28.540	22.79	-38.79		4.10
14	53.899	-28.56	44.37		54.172	-28.96	41.55		1.21
15	45.456	65.66	46.94		45.354	66.50	40.98		2.91
16	86.112	20.98	78.88		83.210	20.63	91.07		3.56
17	54.968	60.36	4.90		53.954	62.06	3.86		1.18
18	48.794	-20.35	-14.16		48.999	-18.08	-14.89		1.62
19	81.321	17.31	25.23		95.471	18.42	21.42		9.40
20	85.753	16.41	16.50		80.967	16.28	18.11		3.39
21	74.915	11.41	17.08		66.381	13.69	15.19		7.11
22	54.365	10.50	13.63		52.181	11.35	12.53		2.50
23	35.330	2.51	12.08		36.449	8.42	9.22		7.90
24	21.295	12.34	5.77		21.375	6.10	5.99		5.96
								average	4.07

Figure S7. Chromatic difference diagram of measured spectrum and simulated spectrum of 24 color blocks.

S2. Evaluation Metrics

In deep learning, the confusion matrix (Confusion matrix) is often used as an evaluation. The confusion matrix integrates the predicted classification and the actual classification, and then separates True positive (TP), False positive (FP), False negative (FN) and True Negative (TN) four categories according to these four categories to calculate the indicators, including Accuracy, Precision, Recall (Sensitivity), Specificity and F1-score.

Accuracy is one of the most common evaluations. By comparing the results predicted by the calculation model with the real value, as shown in Formula 2-1, this indicator can be used to see the training results most intuitively.

$$Accuracy = \frac{TP + TN}{Total\ number} \tag{2-1}$$

Precision refers to the actual accuracy when the prediction is positive. This indicator can be used to identify whether the predicted result is meaningful, as shown in Formula 2-2.

$$Precision = \frac{TP}{TP + FP} \tag{2-2}$$

Recall (Sensitivity) is to see how many actual positive answers can be predicted under the actual positive situation. As shown in formula 2-3, this indicator shows that the number of false positives is very important for detection.

$$Recall = \frac{TP}{TP + FN} \tag{2-3}$$

Specificity is to see how many actual reverse answers can be predicted under the actual reverse situation. As shown in formula 2-4, this indicator is equivalent to Recall, and the number of false negatives can be seen.

$$Specificity = \frac{TN}{FP + TN} \tag{2-4}$$

F1-score is the harmonic mean of Precision and Recall, as shown in Formula 2-5. In extreme cases, both indicators are bad. Precision may hardly predict named entities, and Recall may make too many prediction errors. At this time, F1 Score is an indicator that can reconcile the two.

$$F1 - score = \frac{2}{\frac{1}{Precision} + \frac{1}{Recall}} \tag{2-5}$$

S3. Data Partitioning and Validation Strategy

Before training the neural network model, the dataset needs to be divided into a training set and a test set. There are a total of 3256 images related to dementia, including 1254 normal images, 1320 images of mild cognitive impairment (MCI), and 682 images of dementia, as shown in Table S1. The training set uses 1003 normal images, 1056 Mci images, and 546 images of dementia, while the test set uses 251 normal images, 264 Mci images, and 136 images of dementia, as shown in Table S2.

Table S1. Quantity of Imaging Data for Dementia.

	Classification	Number of images (photos)	Total
Dementia	Normal	1254	3256
	Mci	1320	
	Dementia	682	

Table S2. Classification of Imaging Data in Dementia.

		Classification	Number of images (photos)	Total
Dementia	Training set	Normal	1003	2605
		Mci	1056	
		Dementia	546	
	Test set	Normal	251	651
		Mci	264	
		Dementia	136	

This study randomly divided the dataset into training and testing sets, and set a training period of 50 epochs. We evaluated the learning stability of the models by monitoring the convergence of the loss function, and quantified the classification performance using metrics such as accuracy, precision, recall, and F1-score. The following is a detailed analysis of each model:

1. ResNet50 Model Analysis (corresponding to Figure S8 and Table S3)

Training Convergence (Loss Curve):

Figure S8 shows that ResNet50's loss decreases rapidly in the early stages of training, then flattens out after approximately the 20th epoch.ORI (Original Image): The training and validation curves converge well, but the final loss value is slightly higher than HSI.HSI (Hyperspectral Image): The HSI loss curve decreases more significantly and is more stable, indicating that the model can learn from spectral features more effectively. The validation curve closely follows the training curve, proving that the model does not experience significant overfitting.

Performance Metrics:

According to Table S3, ResNet50 performs best on the HSI dataset, achieving an overall accuracy of 84%, significantly better than ORI's 80%. This indicates that the residual network structure can effectively extract deep features from hyperspectral images.

2. Inception_v3 Model Analysis (Corresponding Figure S9 and Table S4)
Training Convergence (Loss Curve):
Figure S9 shows that the convergence process of Inception_v3 is relatively oscillating, which may be related to its relatively wide network structure. Both ORI and HSI eventually converge to a stable value in terms of loss curve. It is worth noting that the convergence speed of HSI is comparable to that of ORI, and it does not show a significant advantage in accelerating convergence.
Performance Metrics:
As shown in Table S4, Inception_v3 performs similarly on both datasets, with an accuracy of 80% for both. This indicates that for the Inception_v3 architecture, the additional spectral information does not bring a significant gain in classification accuracy, but the model still maintains stable predictive ability.
3. GoogLeNet Model Analysis (Corresponding Figure S10 and Table S5)
Training Convergence (Loss Curve):
Figure S10 shows that GoogLeNet exhibits a very smooth convergence curve.
HSI Advantages: Compared to ORI, HSI's validation loss curve remains at a lower level with less fluctuation, suggesting that hyperspectral information helps improve the model's generalization ability to unseen data.
Performance Metrics:
Table S5 shows that GoogLeNet's accuracy on HSI improves to 83%, better than ORI's 81%. Its Inception module structure demonstrates good adaptability when handling spectral features.
4. EfficientNet Model Analysis (corresponding to Figure S11 and Table S6)
Training Convergence (Loss Curve):
In Figure S11, EfficientNet's loss decreases extremely rapidly in the early stages of training.
HSI Advantages: HSI's loss curve is more stable and lower than ORI in the later stages, showing that the Compound Scaling method, combined with spectral information, can more accurately capture retinal lesion features.
Performance Metrics:
According to Table S6, EfficientNet achieves an accuracy of 82% on HSI, superior to ORI's 80%. Despite having fewer parameters, it still achieves competitive classification performance with the aid of hyperspectral imagery.
Validation of Effectiveness: The loss curves of all models show synchronous convergence on both the training and validation sets, with a small gap between them, demonstrating that our proposed classifier has good generalization ability and no severe overfitting.
Hyperspectral Advantages: Except for Inception_v3, the other three models (ResNet50, GoogLeNet, EfficientNet) show lower loss convergence values and a 2% to 4% improvement in final accuracy after using HSI data. This confirms that incorporating

spectral information can effectively assist deep learning models in identifying dementia features.

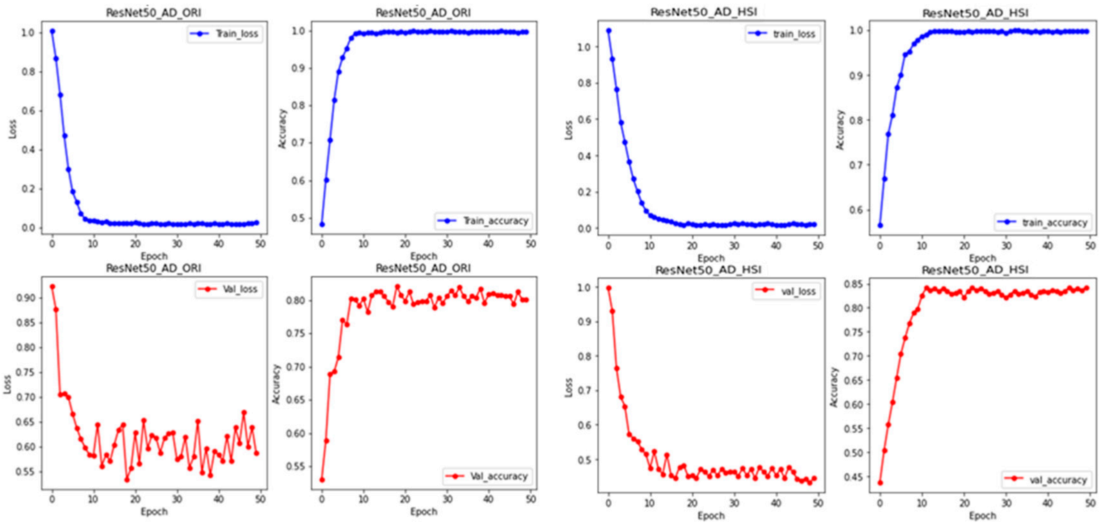

Figure S8. shows the Loss curve and accuracy graph (ResNet50).

Table S3. Dementia Assessment Criteria Results Table (ResNet50).

ResNet50					
AD_ORI	Accuracy	Precision	Recall	Specificity	F1-score
Dementia	0.80	0.73	0.88	0.90	0.80
Mci		0.87	0.63	0.94	0.73
Normal		0.79	0.93	0.83	0.86
Mean		0.80	0.82	0.89	0.80
ResNet50					
AD_HSI	Accuracy	Precision	Recall	Specificity	F1-score
Dementia	0.84	0.78	0.78	0.94	0.78
Mci		0.84	0.81	0.89	0.83
Normal		0.87	0.90	0.90	0.88
Mean		0.83	0.83	0.91	0.83

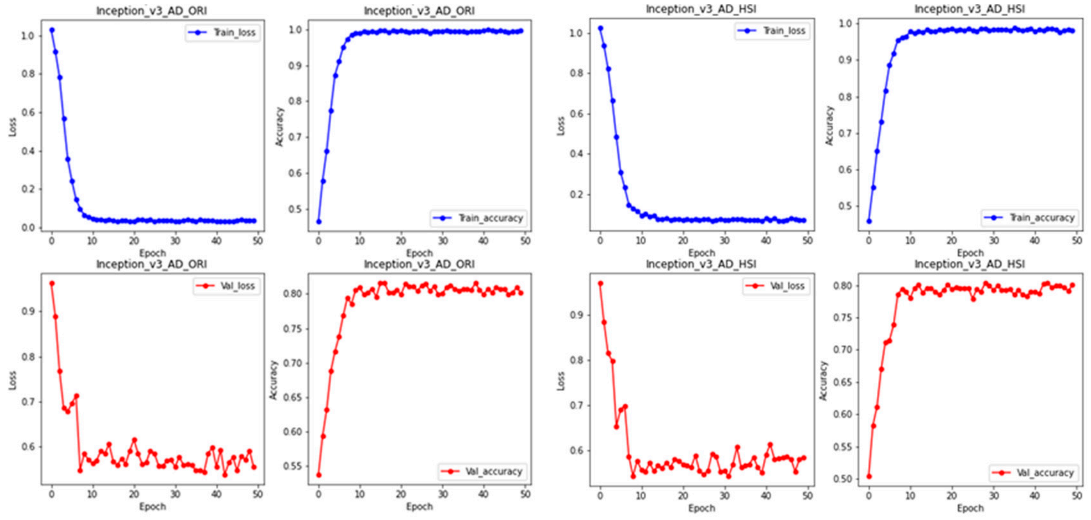

Figure S9. Loss curve and accuracy plot (Inception_v3).

Table S4. Dementia Assessment Criteria Results Table (Inception_v3).

Inception_v3					
AD_ORI	Accuracy	Precision	Recall	Specificity	F1-score
Dementia	0.80	0.69	0.77	0.90	0.73
Mci		0.86	0.69	0.92	0.76
Normal		0.82	0.94	0.85	0.88
Mean		0.79	0.80	0.89	0.79
Inception_v3					
AD_HSI	Accuracy	Precision	Recall	Specificity	F1-score
Dementia	0.80	0.67	0.83	0.88	0.74
Mci		0.85	0.70	0.91	0.77
Normal		0.84	0.89	0.88	0.87
Mean		0.79	0.81	0.89	0.79

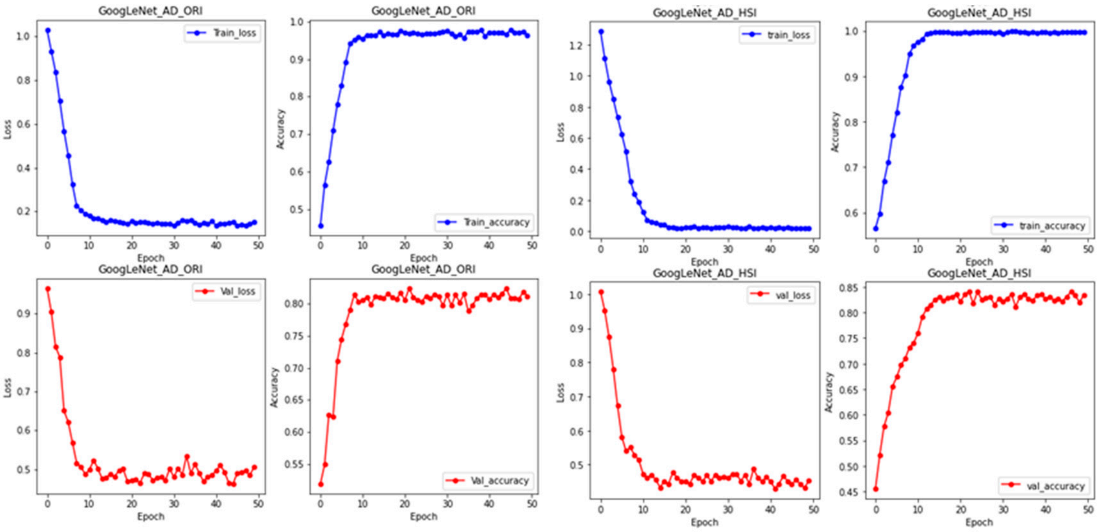

Figure S10. Loss curve and accuracy plot (GoogLeNet).

Table S5. Dementia Assessment Criteria Results Table (GoogLeNet).

GoogLeNet					
AD_ORI	Accuracy	Precision	Recall	Specificity	F1-score
Dementia	0.81	0.78	0.81	0.93	0.79
Mci		0.88	0.68	0.93	0.77
Normal		0.78	0.96	0.81	0.86
Mean		0.81	0.81	0.89	0.81
GoogLeNet					
AD_HSI	Accuracy	Precision	Recall	Specificity	F1-score
Dementia	0.83	0.79	0.79	0.94	0.79
Mci		0.86	0.78	0.91	0.82
Normal		0.83	0.91	0.87	0.87
Mean		0.83	0.83	0.91	0.83

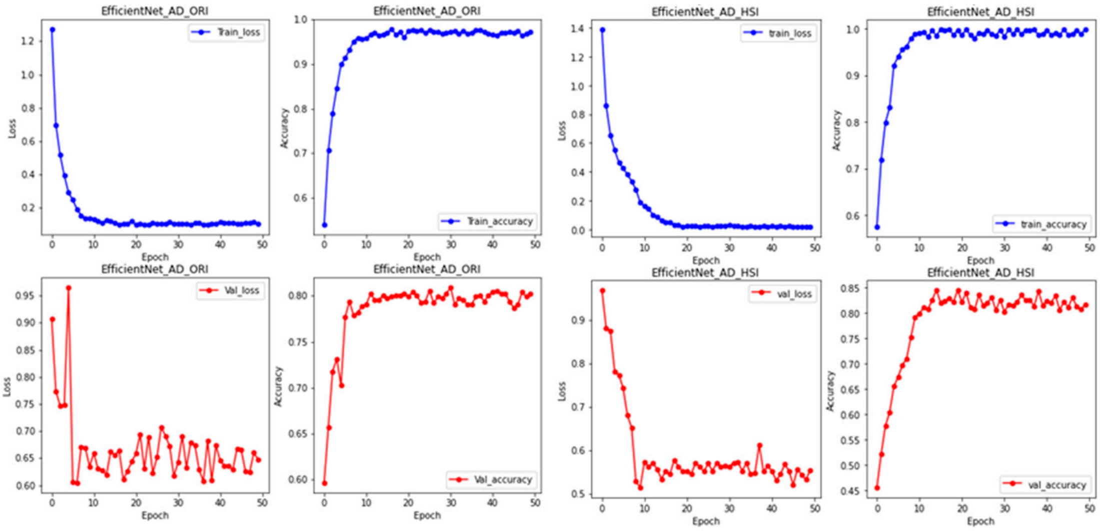

Figure S11. Loss curve and accuracy plot (EfficientNet).

Table S6. Dementia Assessment Criteria Results Table (EfficientNet).

EfficientNet					
AD_ORI	Accuracy	Precision	Recall	Specificity	F1-score
Dementia	0.80	0.73	0.81	0.91	0.77
Mci		0.89	0.65	0.94	0.75
Normal		0.78	0.96	0.81	0.86
Mean		0.80	0.81	0.89	0.79
EfficientNet					
AD_HSI	Accuracy	Precision	Recall	Specificity	F1-score
Dementia	0.82	0.71	0.88	0.89	0.78
Mci		0.87	0.71	0.92	0.78
Normal		0.85	0.89	0.88	0.87
Mean		0.81	0.83	0.90	0.81

Disclaimer/Publisher's Note: The statements, opinions and data contained in all publications are solely those of the individual author(s) and contributor(s) and not of MDPI and/or the editor(s). MDPI and/or the editor(s) disclaim responsibility for any injury to people or property resulting from any ideas, methods, instructions or products referred to in the content.
